# Norwegian pre-service teacher students’ and public health nursing students’ views on health – a qualitative study of students’ perceptions

**DOI:** 10.1080/17482631.2024.2322705

**Published:** 2024-03-03

**Authors:** Turid Kristin Bigum Sundar, Hanna Sargenius, Lisbeth Gravdal Kvarme, Bente Sparboe-Nilsen

**Affiliations:** a Faculty of Health Sciences, Department of Nursing and Health Promotion, Oslo Metropolitan University, Oslo, Norway; b Department of Psychology, section of cognition and neurosciences, University of Oslo, Oslo, Norway; c Faculty of Medicine and Health, Örebro University, Örebro, Sweden

**Keywords:** Health, life-skills, curriculum, public health nursing students, teacher students

## Abstract

**Purpose:**

In 2020, the Norwegian school curriculum was revised, introducing a new cross-curricular subject, Public Health, and Life Skills. The curriculum emphasizes collaboration between teachers and the school health service. Subsequently, a research project, Literacies for Health and Life Skills, was initiated at Oslo Metropolitan University. The aim was to develop a new approach to the subject. A part of the research was to explore perceptions about good and poor health among teacher students and public health nursing students.

**Methods:**

This study has a qualitative design using auto-photography, group discussions and photo-elicitation interviews as methods to explore the students’ views on health.

**Results:**

A analysis revealed three themes about good health in both student groups: Relaxation and tranquillity, belonging and relations, and enjoyment as important to health. Three themes about poor health emerged in both student groups: The ideal body and self-perception, you are as healthy as you feel, and the best in life is also the worst. The students’ statements were characterized by underlying assumptions about health in society, with a focus on “healthism”. No major differences between the student groups were found.

**Conclusion:**

This study serves as a step towards increased understanding of health perceptions among future professionals working with children and adolescents.

## Introduction

A society that facilitates healthy choices for individuals is of great importance to public health (Benninger & Savahl, 2017; Magdalena, 2015; Norwegian Directorate for Education and Training, 2021a). In recent years, research on children and youths in Norway has shown an increase in self-reported mental health problems, such as symptoms of anxiety, depression, and body dissatisfaction, especially among middle-class girls (Eriksen, 2021, 2022). The same is shown in other western countries (Collishaw, 2015; Högberg et al., 2020). The childhood and youth period is particularly crucial for the development of fundamental cognitive, physical and emotional processes and health-related behaviours and skills (Benninger & Savahl, 2017; Bröder et al., 2017; Magdalena, 2015). During childhood, the most important contributors to children’s and young people’s understanding of what is needed to achieve good health, are their families, friends, and schools. However, today’s children and adolescents also have access to online health information of all kinds, which in different ways influence their understanding of health (Park & Kwon, 2018; Richards et al., 2015). Based on concerns about the above mentioned trends, showing a negative development in health and well-being among children and youths, the Norwegian government introduced a new cross-curricular subject in 2020; *Public health and life skills* (PHLS), as an interdisciplinary theme, in primary and secondary lower schools (Norwegian Directorate for Education and Training, 2021a). Issues related to health and quality of life should no longer be limited to subjects such as physical education and science but become integrated into all school subjects. The aim of the new curriculum is to strengthen children’s health and life skills, critical thinking and critical approach to areas of information (Norwegian Directorate for Education and Training, 2021a). In the revised curriculum, life skills are defined as a person’s ability to cope with life through the ability to understand and influence factors that are important for having a good life (Norwegian Directorate for Education and Training, 2021a). Current topics within PHLS are physical and mental health, lifestyle choices, sexuality and gender, drugs, use of media, consumption, and economy. The meaning of life, interpersonal relationships, the ability to handle thoughts and feeling, to set boundaries and respect others, are also part of the subject (Norwegian Directorate for Education and Training, 2021a). Research has shown that programmes emphasizing social and emotional learning, as well as critical literacy may lead to improvements in children’s well-being, quality of life and health (Fleary et al., 2018; Greenberg et al., 2017).

The school health service in Norway is an agent in schools that children and adolescents have access to. Norwegian law requires that all children and adolescents in schools have access to the school health service (The Norwegian Act on Municipal Health and Care Services, 2011). Public health nurses are the main responsible personnel for the service. Collaboration with teachers on health education, health promotion and follow up regarding mental and physical health challenges among pupils are vital aspects of the public health nurse’s practice (Bjørnsen et al., 2019; Dahl & Clancy, 2015).

Public health nurses have a better knowledge base and better understanding regarding health through their education. Therefore, they may contribute fruitfully where teachers may fall short. Thus, they may be supportive as regards health promotion and preventive work in the school as a whole as well as individual pupils in need of follow up (Bjørnsen et al., 2019).

Studies show some challenges in the collaboration between the school health services and the schools (Federici et al., 2020; Granrud et al., 2019; Helleve et al., 2022). Public health nurses in schools are not employed by the schools, but by the municipalities, and are governed by different legislation than the teachers. Teachers are not required to cooperate with the public health nurses about teaching. Resources that are allocated to the school health services vary considerably between municipalities, however a cross-sectional collaboration between teachers and the school health service is both expected and important (Helleve et al., 2022).

There are some concerns associated with implementing PHLS in schools as to how health is understood by teachers and public health nurses (The Norwegian Directorate of Health, 2010). There is also a lack of understanding about the potential for increasing inter-professional collaboration concerning the new curriculum. How the future public health nurses and teachers understand and identify health issues and follow up on the students, may be decisive for how the pupils in turn influence their own health. The question is how to empower students to influence their own health, without this becoming a new way of institutionalizing identity formation, but rather becoming a change in the current discourse, and in that way can become a resource for young people in their pursuit of self -identity (Riese et al., 2022).

Public health nursing education is a one-year postgraduate program-for nurses.^1^ The course is based on a salutogenic understanding of health, with a focus on people’s resources and factors that promote health rather than risk-factors and disease (Laholt et al., 2022; Lindström & Eriksson, 2010; Povlsen & Borup, 2011). During the postgraduate course at the university, the public health nursing students should develop the ability to critically assess factors related to good or poor health, including factors in society that may have an impact on people’s health (Samdal, 2021). However, little is known about how public health nurses understand health, how they work to support child and adolescent health within the school system, and how they cooperate with teachers on health education (Morberg et al., 2006).

The Norwegian teacher education is a five-year programme leading to a master’s degree. The knowledge discourse that seems to dominate the teacher profession is based on values such as development of an individual knowledge base, reflexive practice, and professional autonomy (Karseth & Nerland, 2007). Emphasis is on the individual teacher’s freedom to develop their own personal teaching practice (Nerland & Karseth, 2015). As regards health, studies have shown that physical education often offers a restricted understanding of health as a basis for teaching, with a focus on students’ physical health, rather than taking a more holistic approach (Quennerstedt, 2008). These findings show that the knowledge discourse between teacher and public health nurses appears to differ, which may create challenges in the cooperation between the two groups.

In 2020, based on concerns about how to implement the PHLS cross-curriculum renewal, a project named Literacies for Health and Life Skills (HLS) was launched at Oslo Metropolitan University (OsloMet). The main aim of the project was to develop and implement a new curriculum approach to the PHLS subject. As part of the project, pre-service teacher students and public health nursing students at OsloMet were invited to participate in an inter-professional PHLS week named Health Week, which was held in September 2020. The purpose of the week was to increase the students’ knowledge about youth health and well-being through common lectures, and to introduce them to the use of different visual methods as tools to develop critical health literacy (CHL). This article focuses on the mixed student groups that were asked to use auto-photography and photo-elicitation interviews as methods to elicit their views on either good or poor health (Bugos et al., 2014).

## Aim

The current study aimed to investigate what characterizes public health nursing students’ and pre-service teacher students’ perceptions of either good or poor health, and the differences between the two student groups.

This may provide valuable knowledge about barriers and facilitators regarding cooperation and implementation of PHLS in schools.

## Methods

### Design

Visual methods in social and health research have shown to be well-suited tools for participatory research (Phoenix, 2010). They are creative processes that may enable exploration of how people understand different aspects of their lives and how they make sense of the world (Bugos et al., 2014; Phoenix, 2010). This way of generating data is suggested to be a good method to investigate how people view their lived and experienced world (Harrison, 2002). In this study, we applied a qualitative research technique, based on auto-photography and qualitative photo-elicitation interviews. In photo-elicitation interviews, participants are asked to comment on their own and each other’s photographs, generating further discussion (Schell et al., 2009). Asking participants to take their own photographs forces them to make conscious decisions and actively reflect upon who they are and how they want to portray themselves to others (Schell et al., 2009). Such interviews may evoke feelings, memories, and information that could not be obtained within a traditional interview setting (Harper, 2012). One of the strengths of photo-elicitation interviews, compared with more traditional forms of interviewing, is that they provide participants with the freedom to choose what they want to talk about throughout the interview, which gives them more in control of the situation (Noland, 2006). It is also suggested that those who are less articulate can get support from the other participants when trying to express themselves on a more complex and abstract level (Pain, 2012).

### Participants

Attendance at Health Week was mandatory for all students. A total of 60 Norwegian pre-service teacher students in their third semester of the five-year primary school teacher programme, and 32 public health nursing students in their first semester of the one-year programme for nurses, were asked to participate in the research. Six pre-service teacher students and four public health nursing students chose not to participate, leaving 54 pre-service teacher students (29 women and 25 men) and 28 public health nursing students (27 women and one man) as participants. The public health nursing students were somewhat older than the pre-service teacher students. Applicants to the public health nursing programme must have completed a three-year bachelor’s degree programme in nursing followed by a minimum of one year practice as registered nurses. In total, 80.7% of the PHN students were between 30–45 years, and 83.3% of the pre-service teacher students were between 20–25 years of age.

### Data collection

Data were collected during the mandatory week in September 2020. To emphasize the focus on inter-professional collaboration in the PHLS subject, the participating students were randomly mixed across their professional educations and divided into two main groups, named green and yellow. Those who elected not to be part of the research were placed in two separate groups. Participants in the yellow group were commissioned to take pictures of what they associate with good health, and those in the green group to take pictures of what they associate with poor health. Each student was asked to take three photographs. A few days after the photograph sessions, the students met at the university to discuss their pictures in group interviews. The green group was divided into 10 sub-groups and the yellow group into 11 sub-groups, each with four or five students. The group interview sessions started with the researchers giving an introduction and explanation of the intention of the group interviews to all participants in plenary. The students were given two main group assignments; the first was to present and describe their pictures to their fellow group members, elaborate on why they had taken their pictures and to share how they felt about their pictures. For the second group assignment, students were asked to discuss similarities or differences in their pictures, to look for recurring motives and to discuss which understanding of health was expressed. Throughout the interview session, every student was encouraged to ask a minimum of one question of their fellow group members related to the pictures described. After the group interview session, and in accordance with the directives set by the research team, the students were given the opportunity to discuss more freely. All interviews were audio-recorded and transcribed verbatim.

### Analysis

The transcripts were analysed using thematic content analysis (Graneheim & Lundman, 2004). A stepwise procedure containing six steps, as described by Glaw (Glaw et al., 2017), was applied through the whole process, with transcription of the interviews as the first step. In step two, the photographs were organized by linking each photograph to the transcribed interviews for each student group, followed by allocating the photographs and transcriptions into main categories of sources for good health, and sources for poor health. During step three, the photographs with the written descriptors were compared, and the most common themes and the less important ones were identified within each main group (good or poor health). In step four, the most frequently appearing themes of motives were identified in the photographs and meaningful quotes were extracted from the transcripts. These were then compared within the green and yellow groups, allowing common themes within each group to emerge. During step five, the final analysis of the patterns and meanings that emerged from the data was clarified and discussed by the research team, by moving back and forth again and again through the photographs and the interview transcripts. In step six, the thematic analysis continued through discovering important themes and connecting them into major/overarching or related themes.

The research team was initially five professors at OsloMet, two from teacher education, and three from the public health nurse education. All five participated in the data collection. The researchers formed two teams, to follow up the students in the green and yellow groups. One from the teacher education did not participate in the proceeding work. Four of the team members continued with interpretation and discussion of data. To ensure trustworthiness, the researchers read an interpreted data from all groups individually, followed by discussions collectively. To prevent preconceptions, preliminary themes were discussed between the researchers. It was important to obtain different perspectives, opinions, and interpretations of the data. Based on these discussions, consensus about the main themes were reached (Koch et al., 2014; Kvale & Brinkmann, 2009).

### Ethics approval

The research project was preregistered and approved by the Norwegian Centre for Research Data (NSD/SIKT project nr. 74915/301598) and was carried out in accordance with their research ethics guidelines. The University of Oslo’s TSD 2.0 system was applied for secure storage of sensitive data. Prior to Health Week, both student groups received written and verbal information about the purpose of the research project, and that it was voluntary to participate. Participants gave a written consent on participation in the study. They were informed that absence of participation in the research project would not have any consequences for them as students. Student submissions via digital portals were anonymous and could not be traced back to the individual IP address.

## Results

**Figure 1.**
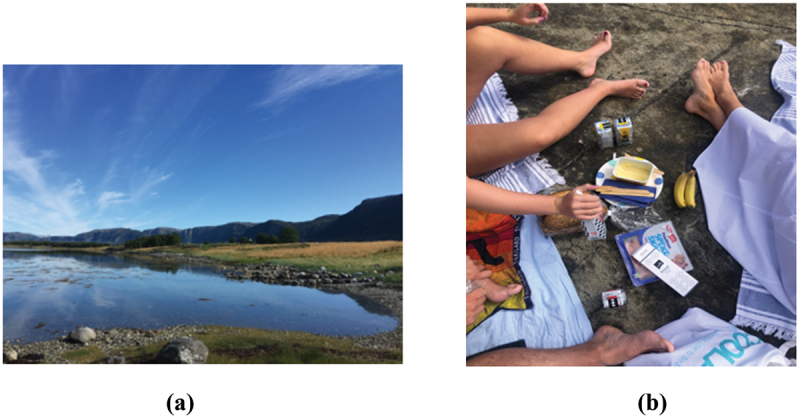
Examples of pictures from the good health group. (a) public health nursing student. (b) pre-service teacher student.

Analysis of the pictures and the transcribed text revealed that the groups assigned to take pictures of good health had mainly chosen images that illustrated different forms of physical activities, nature, relaxation, animals, family, and friends (Figure 1). The emotions described in connection to their pictures were feelings of freedom, calmness, enjoyment, safety, and belonging.

The pictures from the pre-service teacher students and the public health nursing students were similar, except that the public health nursing students had more pictures of relaxation and the pre-service teacher students had more of physical activity.

**Figure 2.**
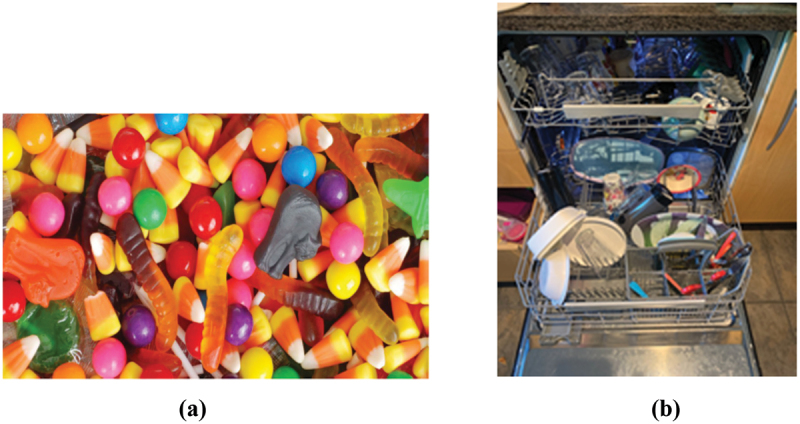
Examples of pictures from the poor health group. **(a)** pre-service teacher student. **(b)** public health nursing student.

Pictures taken by the group addressing poor health included images of non-healthy food, alcohol and tobacco use, untidiness and chaos in their homes, bills, bad sleep, mobile phones, computers, and pictures of slim bodies (Figure 2). The emotions described by these students were feelings of stress, anxiety, pressure to succeed in life, pressure to be fit and slim, and a guilty conscience. Students from both educational backgrounds expressed that the images of unhealthy food, sweets, and alcohol also created feelings of pleasure.

Within both the yellow and the green groups, differences in the type of pictures taken by the pre-service teacher students and the public health nursing students were observed. However, these differences were mainly related to the students’ heterogeneity in age and life situation. The pre-service teacher students had more images related to social media use, partying, and friends, while the public health nursing students had more pictures related to family life.

Analysis revealed three main themes among the good health groups, namely (1): relaxation and tranquillity (2), belonging and relations, and (3) enjoyment important to health.

Within the poor health groups, analysis revealed three main themes, namely (1): the ideal body and self-perception, 2) you are as healthy as you feel, and 3) the best thing in life is also the worst.

## Discussions in the good health group interviews

### Relaxation and tranquillity

Students from both educational backgrounds mentioned physical activity (PA) as important to maintaining good health, both to keep the physical body healthy, and as a means to improve mental health through releasing pressure and reducing stressors. In their descriptions, many of the students also included nature and outdoor life activities as important for good mental and physical health:

The image here really represents nature and exercise at the same time because I exercise … exercise is a very big part of my life, and it’s like, training has in a way become a routine for me then … I get out into the nature, my mind gets aired, and yes, really a lot of that.

(Pre-service teacher student)

Some of the participants emphasized challenges and ambivalence towards PA, but also how the good feelings overshadowed the negative feelings:

I am not really fond of running … or I think that … it can be challenging to go out running, but … the days I’ve been out running at the beginning of the day I feel so much better. So, it’s a very good start to the day when I’ve been out running. It’s so worth it. And I think, it has a physical gain of course, but also somehow in relation to the mental health then, that it is a very good start of the day to feel that both … yes both for the mood and for the body to have started the day with a run or walk.

(Pre-service teacher student)

However, the concept of being physically active outdoors was described by some of the students as being somewhat independent of exercise or the intention of getting fitter. In particular, the public health nursing students mentioned spending time outdoors as a way of relaxing and experiencing feelings of peace and calm. This could be obtained by spending time in a hammock in the garden, or by doing gardening or just being outdoors in beautiful scenery:

The feeling, it gives me the feeling of peace and joy I think, to be out in nature and to experience the wonderful, magnificent nature. So, I manage to relax and not think about everything else I have on my mind, being out in the nature, when you see this magnificent nature.

(Public health nursing student)

Outdoor PA was described by some as contributing to life-changing decisions because of the peace of mind it created:

So … this image, why I chose it, it kind of represents, outdoor yoga and what, it kind of gives me, like peace in mind, the pure mental … and also, I don’t know if change is an emotion, but a positive change in life, then. I kind of felt like this was “on the way”, one of the important things which made me to choose this new path and represents a change I made in adulthood.

(Pre-service teacher student)

### Belonging and relations

Both the pre-service teacher students and the public health nursing students emphasized belonging and relations to family and friends as important for good health:

This is a picture from where we have our cottage … It represents health for me because it is a place where you go to relax and recharge your batteries, both physically and mentally. Here there are lots of opportunities, for relaxing, and swimming, and just to have a lot of fun. I have had a cottage there ever since I was a child, so it is the only place for me that somehow represents feeling properly at home, because everyone in my family has moved away to so many different places so I kind of have no place that is home to me anymore, if you follow me … .

(Public health nursing student)

Several of the images and the students’ descriptions were about spending time with family and friends, in combination with the importance of belonging and doing things together:

Then there is a picture of me and my mum … my mum is very important to me … this was in April this year, when we were suddenly locked inside, so everything was a bit crap. Then my mum got me out every day; we are going for a walk, we are going to get out every day, out in the forest, eat good food, and stuff like that. All those things … this was a bit what I was thinking about, with good health. I think, if it had not been for my mum in that period when we were trapped, I would have felt properly sort of, locked inside. So … she’s very important to me.

(Pre-service teacher student)

However, one of the public health nursing students also expressed some ambivalence related to relations:

This is a picture of me in … because, in retrospect I thought … there is no training … this is not a picture of something which gives me good health, I could have taken pictures of vegetables and fruits … however, good health is more than that … I think there is a lot of feelings in these pictures … I could have taken pictures of my family, but, that is … family is … having children is stress … it is also stressful.

(Public health nursing student)

### Balance is important to health

When discussing good health, all the students talked about the importance of understanding health as something more than just depending on healthy eating and exercise:

It feels like … yes in a way, I also feel that much of it has been what … what gives us good health, is simply what gives us a good feeling and good mental health. It may be in a way like … I completely agree with that you should use your body and such, but I thought about it a bit yesterday, that even though it is a pleasure to exercise and such, there is something … there is something special about being able to travel, your family and relationships, and … with the nature and surroundings.

(Pre-service teacher student)

During these discussions, both the pre-service teacher students and the public health nursing students expressed ambiguity about healthy eating and physical activity:

What is positive health, it is quite often a bit the opposite of what we say, a stable and healthy diet, to be social, have many friends, not be lonely, a good night’s sleep … but at the same time for my part it is a bit like, to have good health I must also be able to do a bit like, eat what I want to eat, when I want it, but to do so then I must also move, so that I will not … my body will not get big and I will get a distorted view of my body and that is not good for health either, so it’s … so, it’s all connected, it goes both ways, but you can be a little on … let’s say on a line … to maintain good health as long as you are over the line in some circumstances … then it balances out in a way.

(Public health nursing student)

Further, several of the students from both educational backgrounds agreed that it was also important to allow oneself to eat something unhealthy occasionally:

Even if it is unhealthy, it may give you good health because you like it so much and enjoys it, yes … so.

(Public health nursing student)

Absolutely and it is … it is…to indulge, it is in a way to experience new…that is…to eat…to experience something new, it is also something completely unique and can give an insane joy … And that of eating food with each other with like several others and experiencing it. It is an experience too.

(Pre-service teacher student)

## Discussions in the poor health group interviews

### The ideal body and self-perception

To illustrate poor health, many of the pre-service teacher students showed images taken from Instagram, movies, television, streaming services, mobile phones, Fitbit watches, and apps. Some of the pictures illustrated the use of social media and telephones; others showed pictures of how people use social media to portray themselves. A few of the public health nursing students had taken similar pictures to those of the pre-service teacher students. When analysing the discussions among the different group members, both the pre-service teacher students and the public health nursing students expressed worries about how social media use may influence body image and self-perception:

Well, Instagram is a platform where I follow quite a few unhealthy users then, including supermodels who are super thin, fitness people who have “six packs” and it gives me a very unhealthy…it gives an unhealthy influence in the form that I feel that I may not quite be able to achieve the look that is produced on Instagram by these users.

(Pre-service teacher student)

Further, they discussed how retouching of pictures and pictures of people who have undergone cosmetic surgery may reinforce unrealistic expectations about how to look:

Being happy with your body is, of course, very important, but it is also in a way very important to inform about what is realistic and what is unrealistic in the form of retouching, or whether it is a cosmetic operation, whatever, it may be just unrealistic … expectations … and in a way … social norms then.

(Public health nursing student)

Students from both educational backgrounds also mentioned the potential negative effect such body ideals may have on mental health:

Social media is a proven source for mental health problems whether it is body ideals that are impossible to live up to, there is…. pressure on different types of diets or food, or there are in a way many temptations inside such things as … “I want that thing also”. And it is in a way a bit like a culture of shame … .

(Pre-service teacher student)

All the students expressed concern about the negative effect social media use may have on adolescents’ views of themselves during puberty:

It is generally a bad picture of what is supposed to be normal for young people then, somehow. Yes, it is completely normal to have pimples, of course, it is normal to have strange proportions in a time of life because your body is growing…so it is kind of … it’s very unrealistic for people to think but ok, I as a 15-year-old should look like that.

(Pre-service teacher student)

### Stressors and concerns

Other pictures illustrated what the pre-service teacher students and the public health nursing students expressed as examples of stress. Many mentioned that they felt pressure to do all sorts of activities (e.g., leisure activities, cleaning, work, studies, and time with family and friends), and lack of time, as causes of stress and concern. These feelings of stress and concern were also connected to things that they felt they should not do:

When I look at my pictures, I get a little anxious, to be honest, because I look at things that I should not do … on my second picture … I should not drink too much, [I should] know my limits and alcohol tolerance.

(Pre-service teacher student)

The use of mobile phones and social media, and experiencing the expectation that they should always be available were also expressed as a source of stress:

The mobile phone causes a kind of pressure about always being available and that you always should be online, and everyone should be able to reach you at all times.

(Public health nursing student)

Some pointed out that due to feelings of stress and concern, they were not able to be present in the moment, enjoying what they were doing. Other aspects of stress, such as poor finances, were also highlighted:

When you get an invoice, or a bill, it is not very fun right? All of a sudden it comes … Why I have chosen this exact picture, is because it is probably like that … it is everyday stress like most people probably experience, having to pay bills and it goes on and on.

(Pre-service teacher student)

### The best thing in life is also the worst

Those in the poor health group were aware of the importance of having a balanced and healthy diet, moderate alcohol consumption, and avoiding the use of tobacco to maintain good health. However, they also linked unhealthy food and alcohol consumption to “enjoyment”, and the ability to having a good time. Several stated that it may be harmful to think about health all the time:

It is important to point out … chips and such things, it does not have to be harmful to health, but it is about the amount of it and if you replace it with a meal and if you are overeating it.

(Pre-service teacher student)

It may be a bit like, yes alcohol is not good for you, but if you take a glass of wine, like occasionally and not too often, then it does not matter. But if you do take a drink every weekend, Friday, Saturday, every week, then, yes, then it is not so good for your health (…). Nonetheless, it is true for the most part that nothing is directly unhealthy in small, limited doses. It is about the extent and how much you use things and take drinks and eat that determines how unhealthy it becomes. Everything can represent ill health if you just do enough, like training too much or eating too little … .

(Pre-service teacher student)

Even though the participants expressed that a healthy diet was important to health, they stated that they often found it easier to choose the less healthy food alternatives. This was both due to easy accessibility and the taste of the food (sweet, salty, or fatty). Sweets and fast food were cited as examples of food that one enjoys there and then but which may be a source of negative emotions from a guilty conscience afterwards:

This picture shows a drawer with sweets. It is actually me who buys the stuff, but it is very tempting to take some candies occasionally … it gives me pleasure in a way, but it is also…I know that it is not good to eat too much of it.

(Public health nursing student)

I really, really love chocolate and I get happy from [eating] chocolate and maybe … not really, it’s not that I associate it with something bad really, but it’s maybe an unhealthy thing if eating too much chocolate even though you become happy when eating it.

(Pre-service teacher student)

When smoking and snuff use were mentioned, all participants were unanimous in their position on the negative effects, even though one student who was a snuff user also highlighted positive elements:

Research shows that it [tobacco and snuff] can lead to cancer of the oral cavity, and it is addictive, which is bad for health and your fitness level is getting reduced. The feeling I get [using it] is also a feeling of guilty conscience, but also that there is something that gives you a false sense of security in that you become calm in a way.

(Pre-service teacher student)

## General discussions within the yellow and green groups

### You are as healthy as you feel

A few subgroups within both the yellow and green groups went beyond discussing the assignment topic of what good or poor health meant to them. The students’ discussion focused on the concept of health in general, but also on how individuals may experience health differently:

What we might be talking about is “what is poor health”? Is this bad health for me? Or is it bad health for others? Because what we are talking about in my profession is that you are as healthy as you feel, and even though I have cancer, I can feel healthy, right? So, what is disease to me is not necessarily disease to you. So, you should not impose on others your own views on health.

(Public health nursing student)

Exercise was used as an example of how different views on health may be based on the individual’s own standpoints:

Yes, it’s like that when it comes to exercising. For example, those who exercise look at it as unhealthy not to exercise, while those who do not exercise do not usually walk around and think that “shit, I have poor health because today I have not worked out”.

(Pre-service teacher student)

Their roles as professionals with the assignment of promoting health, and the ambiguity between freedom of choice and responsibility were also discussed:

Yes, there is a choice. And there is a choice in what you can say about health then, do you have a choice when it comes to health? If you can choose your own good health, to a certain extent you can. (…) So, you can create the conditions to not get cancer by not sniffing, but then…(…) So, then there is something about not giving people a bad conscience in the professional role that we have. You should not inflict on people a guilty conscience because people manage to inflict a guilty conscience on themselves. So, we should be the last to create a guilty conscience, although it is a requirement that we must inform those who do not understand, because there are a bunch of people who do not understand out there.

(Public health nursing student)

In that sense, I think we all have an opportunity to remove what gives us poor health. For my part, it’s like “yes, it’s a stressful study situation, but it disappears by Christmas, because then I’m done with my studies.” So, there is that it is not forever, it is not forever. And maybe then it will also be easier to carry this “poor health” then, when you know that you have the guarantee that it will pass away. And for our part, all those things go away in one way or another.

(pre-service teacher student)

The students from both the good and the poor health groups discussed their experiences of using photo-elicitation as a basis for talking about health. Several mentioned that they saw it as a potentially useful tool in their future work in schools. Students from both groups also highlighted that the Health Week, and the discussions, had given them an increased and valuable insight into each other’s professional backgrounds, considering future collaboration.

## Discussion

This study served as a step towards a better understanding of the health perceptions of those training to work with children and adolescents in the future, namely public health nursing students and pre-service teacher students. The results of our analysis revealed three main themes among the good health student groups (1): relaxation and tranquillity (2), belonging and relations, and (3) enjoyment is important to health. The three main themes among the poor health student groups were: 1) the ideal body and self-perception, 2) you are as healthy as you feel, and 3) the best thing in life is also the worst. Several of the students from both the good and the poor health groups elevated the discussion to the concept of health in general and to their role as future cooperating professionals working in schools. During these discussions, the value of getting to know each other’s functions, responsibilities, and potential cooperation related to health education were emphasized. The potential of using photo-elicitation as a tool in health education was also discussed.

In recent decades, there has been a significant increase in the focus on health, and the importance of health in everyday life (Crawford, 2006). Protecting and securing health has increasingly become an obligation for societal institutions, as shown by the new curriculum (Crawford, 2006). However, health as a concept is complex. Traditionally, the term “health” is often dichotomized into good health and poor health, while illness is perceived as a medically understood and empirically proven deviation from the health norm and is something that you either have or do not have. In line with this biomedical assumption that health is either good or poor, various health measures have been aimed at educating the population, to protect them from and reduce the risk of what can be defined as poor health (Lindström & Eriksson, 2011). Studies have shown that a traditional approach to health as good or poor does not necessarily have any effect on health behaviours but rather may evoke compensatory unhealthy behaviours (Sim & Cheon, 2019; Werle et al., 2011). This is supported by research on smokers and drinkers, which shows that their attempts to limit use led to more, rather than less, use (Dohle & Hofmann, 2019; Maloney et al., 2021; Muraven et al., 2005). What complicates health further is that health behaviours do not happen in isolation, but are connected to people’s social networks, friendships, socioeconomic status, access to food, social media, and body ideals in society (Lightfoot et al., 2019).

Similar to the findings of other studies, the participants in this study expressed that they considered health to be in the context of what contributes to good health or not, and not merely the absence of disease (Farre & Rapley, 2017; WHO. World Health Organization, 1948). As such, the students from both the good health and the poor health groups went beyond the World Health Organization’s 1948 definition of health, which has been criticized for its absoluteness in using the word “complete” in relation to health and well-being (Huber et al., 2011). In that sense, the students expressed what can be described as a more holistic and critical understanding of health, with broad and complex perceptions of what lies in the concept (Fugelli & Ingstad, 2014). We could not find any distinct differences between the two student groups when discussing these issues, or between the good health and the poor health groups. The latter is possibly due to the fact the students had taken part in a common Health Week.

The students’ discussion of good health highlighted belonging, nature, and the importance of other factors in life, such as “enjoyment”, as being important to health. Most students made a strong connection between social contact, belonging, and health. Belonging was related to people and places that were essential to them. This is in accordance with belongingness theory, which emphasizes the need to belong as being important to people’s cognitions, emotions, behaviours, and overall health (Allen et al., 2022; Baumeister & Leary, 1995). Further, the participants expressed a view on health that was more than just having good physical health. What promotes good health is what makes one feel good, with a balance between the healthy and unhealthy. Similar to findings in other research, the participants conceptualized the tension between pleasure and health shown in Western societies (Jallinoja et al., 2010). According to Crawford, this may be due to the cultural ethics of work and discipline, which for some time have been joined by an ethic of consumption and fulfilment of desires, emphasizing not only self-control, but also pleasure, and enjoyment as important sources of health (Crawford, 2006). The students from the poor health groups expressed a similar view in their discussions around “the best thing in life is also the worst”. The goal conflict between short-term satisfaction and long-term health considerations that they highlighted is at the heart of what makes it difficult to promote healthy behaviours through health education (Crawford, 2006).

The importance of external factors such as politics and society were not significantly problematized by any of the participants. Most of the students’ statements were characterized by a health discourse of risk avoidance and personal responsibility for their own health (Leahy, 2014). According to Crawford (Crawford, 2006), this individualistic “healthism” approach neglects the complexities of social constructs of health (Fane et al., 2019). Fane et al. (Fane et al., 2019) claim that it is within these contemporary risk-oriented understandings of health that one sees an increased concern regarding health improvement.

Social media use was frequently discussed among the poor health groups in this study. Based on their own experiences with social media, and how they themselves had been influenced by it, the participants expressed worries about such influences on adolescent health and well-being. Pictures illustrating social media use were slightly more common among the pre-service teacher students, possibly because they were younger and more accustomed to digital interactions. One of the challenges that they particularly expressed concern about was the flow of unregulated online health information that may influence young people’s self-image and health in different ways (Meland et al., 2006; Svalastog et al., 2017). Today, knowledge about the content and scope of health, its form and how it becomes accessible through technology, is changing continuously (Svalastog et al., 2017). Health as a topic is no longer only reserved for experts, but has instead become public property (Mæland, 2013). The potentially negative influence of social media on health is supported by other research, which indicates that smartphones and online internet access increase mental distress and self-injurious behaviour, with the greatest impact on girls (Abi-Jaoude et al., 2020). Other studies, however, do not conclude as confidently (Berryman et al., 2018; Heffer et al., 2019). Nevertheless, access to health information through social media and internet use are challenges that potentially may complicate health education further.

The students in this study are in a special position. Both student groups are in professional fields where they will learn through practice and interaction (Hoffman et al., 2015; Horntvedt et al., 2018). How they explain to and teach children and young people about health will be greatly affected by their health competence and how they are able to use the information they acquire with the aim of acting for the benefit of pupils in schools. Children and young people can be vulnerable to the demands of modern society and schools have an important influence on young people’s health and well-being (Littlecott et al., 2018). Research suggests that the culture and values promoted in schools is essential in this regard (Bonell et al., 2013; Langford et al., 2017). Based on the flow of information that young people are exposed to, it seems more important than ever to strengthen their abilities to engage critically about health topics (Diviani et al., 2015). Worldwide, this has led to a change in focus, from a traditional approach to health education, to a strengthening of health literacy as an alternative and crucial goal (Haugen et al., 2022; Paakkari et al., 2012). The Norwegian HPLS curriculum in schools is in line with this development (Norwegian Directorate for Education and Training, 2021a, 2021b). It is still important to have a critical view of the new subject, which is based on risk conceptions about adolescent’s mental and physical health issues, and to critically question the underlying intentions of such programmes (Riese et al., 2022).

Understanding health as a concept is paramount to understanding the challenges in connection with implementing the new curriculum in schools. In many cases, health is subjective, and as the students’ photos of nature, PA, healthy and unhealthy food, social media use, stress, family, and friends show, it is also dependent on other factors. The objective facet of health, as expressed in public health guidelines, do not necessarily conflict with subjective experiences of what gives them good health. Many of their pictures and statements are in accordance with the guidelines. To manage their daily lives, they modify their personal views on health in terms of expressing the necessity to find a balance between “the good and the bad”. The participants’ reflections reveal the complexity of the concept of health.

In view of the findings presented in this article, some educational arguments as regards preparation of both teachers and public health nurses working in schools would likely be beneficial for the schools and pupils as a whole. The public health nurse is normally localized at the schools, with a designated office. Children and youths frequently visit the public health nurse for a variety of reasons. Often, teachers ask the public health nurse to take certain topics such as sex education, health, and hygiene. Taking part together in preparatory courses for the new interdisciplinary subject should increase mutual interprofessional thrust and cooperation, and long-term quality at the schools. Interprofessional cooperation in curriculum building and implementation may also contribute to better cooperation between university faculties (Lindqvist et al., 2019).

The upshot of our findings and discussion may be summarized as a critical need to strengthen the emphasis on health literacy, and health literacy in a mediated world. Today, sources of information are global, and information on health-related aspects of young peoples’ lives are easily accessible. Developing critical competence in health literacy should be a vital task of the school. Teachers and public health nurses also need to develop their health literacy in their professional programmes. By extension this pertains to all institutions of higher learning with education in teaching and public health nursing.

## Strengths and limitations

By approaching our research question with a combination of different qualitative methods, participants in this study were given the opportunity to express their beliefs and ideas in a non-verbal manner in addition to the spoken word. As such, even though the interviews were conducted by the participants themselves, and not by an independent researcher, we believe that these equal and collaborative interactions within the student groups secure the quality of the acquired data. Another strength of this study is that by allowing our participants to choose their own personal images for the photo-elicitation interviews, they had time to think about which photos they wanted to present to their fellow students. It also gave them the opportunity to reflect at a deeper level on the topic of health, prior to the discussion session itself. Hence, it allowed participants to analyse their lives in a way they had not done before, providing valuable insights for themselves and about each other, both as individuals, and as two professional groups that are supposed to work together in the future.

Furthermore, health is a very complex concept to articulate. In the literature, reasons given for choosing a visual method frequently relate to facilitating communication on topics that are difficult to raise either because they are largely subconscious or subject to social or psychological inhibitions (Blackbeard & Lindegger, 2015; Glaw et al., 2017). All in all, the process the pre-service students and public health nursing students went through during Health Week, preparing them for the interviews and working with visual methods, created a richness in the data. The students add additional layers to the data they speak about and are able to analyse their own material, which in turn creates joint theorizing during the photo-elicitation interviews.

A potential limitation of our research is that the researchers may have interpreted the photographs and other visual aids differently to the participants. To minimize this, photo-elicitation interviewing was used as a member checking tool. This is in accordance with the recommendations which state that auto-photography should be used in conjunction with photo-elicitation interviewing to allow participants to explain exactly what the photographs mean so that misunderstandings or misinterpretations of the photographs are minimized (Glaw et al., 2017). Another limitation is that the students were asked to take pictures that represent their opinions of good or poor health, instead of just health as such. The latter may have led to different results, since giving them an assignment to take pictures of good or bad health guided them in a certain direction. Further, there were twice as many pre-service teacher students as public health nursing students, very few of the participants were men, and the pre-service teacher students were, on average, younger than the public health nursing students. This may also have affected the results. This research was conducted in connection with the health week. Doing a similar study independent of this week, may have generated other findings.

## Conclusion

The current study aimed to investigate what characterizes the perceptions of either good or poor health held by public health nursing students and pre-service teacher students, and the differences between the two student groups. Several of the students in this study expressed a certain degree of holism when discussing their pictures of good or poor health. However, many of their statements were also characterized by underlying assumptions of health in society, with a focus on risk and “healthism” (Crawford, 2006). We could not find any major differences between the pre-service teacher students and the public health nursing students. Our participants’ statements show that the future teachers and public health nurses need to broaden their views on health further to accomplish the goals of the new curriculum.

Further research should focus on teachers’ experiences in integrating PHLS in all subjects and address the interprofessional cooperation between the teachers and public health nurses in the work with the new curriculum in schools. In colleges and universities, one should work to institutionalize interprofessional curricular topics on health literacy issues, and research the impact on the pre-service teacher and public health nursing students. In the future, research on the critical health literacy of pupils in the schools should be undertaken to find out whether it increases.

## Supplementary Material

Biographical_note_about_the_authors.docx

## Note

1. From 2022, the public health nursing education became a two-year master’s in health sciences degree, with specialization in public health nursing.
