# Supplementary material for: Norwegian pre-service teacher students’ and public health nursing students’ views on health – a qualitative study of students’ perceptions
Source: Int J Qual Stud Health Well-being. 2024 Mar 3;19(1):2322705. doi: 10.1080/17482631.2024.2322705 (PMC10911246; doi:10.1080/17482631.2024.2322705)
Supplement: Biographical_note_about_the_authors.docx [file ZQHW_A_2322705_SM9970.docx]

***Norwegian pre-service teacher students’ and public health nursing students’ views on health–a qualitative study of students’ perceptions***

**International Journal of Qualitative Studies on Health and well-being**

Turid Kristin Bigum Sundar (corresponding author)

Orcid: <https://orcid.org/0000-0001-7862-9413>

Affiliation: [Faculty of Health Sciences](https://www.oslomet.no/en/about/employee-directory?unitcode=hv), [Department of Nursing and Health Promotion](https://www.oslomet.no/en/about/employee-directory?unitcode=hv&sectioncode=hv-u-sha), Oslo Metropolitan University, Norway.

Turid Kristin Bigum Sundar has a PhD from the University of Oslo, Norway. She conducted her PhD on school children with overweight and their experiences with participation in an internet-based intervention to increase physical activity, improve physical fitness and quality of life. She is employed as an associate professor at the master’s degree programme in health sciences, with specialization in public health nursing, at the [Faculty of Health Sciences](https://www.oslomet.no/en/about/employee-directory?unitcode=hv), [Department of Nursing and Health Promotion](https://www.oslomet.no/en/about/employee-directory?unitcode=hv&sectioncode=hv-u-sha), Oslo Metropolitan University, Norway. Her research focus is on school children, overweight and physical activity interventions, quality of life, well-being and health care, and public health nursing students’ clinical practice.

Hanna Sargénius

Orcid: <https://orcid.org/0000-0002-4502-9443>

Affiliation: Department of Psychology, section of cognition and neurosciences, University of Oslo, Norway.

Hanna L. Sargénius has a PhD in psychology and is a post-doctoral researcher at the Department of Psychology, section of cognition and neurosciences, University of Oslo, Norway. She conducted her doctoral studies on medical interventions of morbid obesity and neuropsychological functioning at the Norwegian University of Science and Technology, Trondheim. Her research focus today is cognitive interventions for improving executive functions and fatigue in pediatric acquired brain injury and explore long-term quality of life outcomes.

c

Orcid: <https://orcid.org/0000-0002-8779-7512>

Affiliation: [Faculty of Health Sciences](https://www.oslomet.no/en/about/employee-directory?unitcode=hv), [Department of Nursing and Health Promotion](https://www.oslomet.no/en/about/employee-directory?unitcode=hv&sectioncode=hv-u-sha), Oslo Metropolitan University, Norway.

Lisbeth Gravdal Kvarme has a PhD from the University of Bergen, Norway. She conducted her PhD on self-efficacy and health-related quality of life in socially vulnerable school children. She is employed as professor at the master’s degree programme in health sciences, with specialization in public health nursing, at [Faculty of Health Sciences](https://www.oslomet.no/en/about/employee-directory?unitcode=hv), [Department of Nursing and Health Promotion](https://www.oslomet.no/en/about/employee-directory?unitcode=hv&sectioncode=hv-u-sha), Oslo Metropolitan University, Norway. Her research focus is on vulnerable school children, bullying, quality of life and self-efficacy.

Bente Sparboe-Nilsen

Orcid: <https://orcid.org/0000-0002-8983-812X>

Affiliation: [Faculty of Health Sciences](https://www.oslomet.no/en/about/employee-directory?unitcode=hv), [Department of Nursing and Health Promotion](https://www.oslomet.no/en/about/employee-directory?unitcode=hv&sectioncode=hv-u-sha), Oslo Metropolitan University, Norway. Faculty of Medicine and Health, Örebro University, Sweden.

Bente Sparboe-Nilsen has a PhD from Örebro University in Sweden. She conducted her doctoral studies on growth among schoolchildren at Faculty of Business, Science and Engineering, Örebro University, Sweden. She is employed as an associate professor at the master’s degree programme in health sciences, with specialization in public health nursing, at the [Faculty of Health Sciences](https://www.oslomet.no/en/about/employee-directory?unitcode=hv), [Department of Nursing and Health Promotion](https://www.oslomet.no/en/about/employee-directory?unitcode=hv&sectioncode=hv-u-sha) at Oslo Metropolitan University, Norway. Her research focusses primary today on deviant growth patters such as overweight and underweight among children, pedagogic and child abuse.
